# Central histopathological review of a European hepatocellular carcinoma cohort: impact of the WHO 2019 classification on histological diagnosis and TNM staging

**DOI:** 10.1007/s00428-025-04301-4

**Published:** 2025-10-24

**Authors:** Konstantina Dimopoulou, Despoina Myoteri, John Contis, Panagis Lykoudis, Constantinos Nastos, Georgios Fragulidis, Antonios Vezakis, Dionysios Dellaportas, Manousos Konstadoulakis, Ioannis G. Panayiotides, Nikolaos Arkadopoulos, Periklis G. Foukas, Dina Tiniakos

**Affiliations:** 1 Department of Gastroenterology, Red Cross Hospital, Athens, Greece; 2 Department of Pathology, Aretaieion Hospital, Medical School, National and Kapodistrian University of Athens, Athens, Greece; 3 2nd Department of Surgery, Aretaieion Hospital, Medical School, National and Kapodistrian University of Athens, Athens, Greece; 4 3rd Department of Surgery, Attikon Hospital, Medical School, National and Kapodistrian University of Athens, Athens, Greece; 5 4th Department of Surgery, Medical School, Attikon University Hospital, National and Kapodistrian University of Athens, Athens, Greece; 6 2nd Department of Pathology, Medical School, Attikon University Hospital, National and Kapodistrian University of Athens, Athens, Greece; 7 Translational and Clinical Research Institute, Faculty of Medical Sciences, Newcastle University, Newcastle Upon Tyne, UK

**Keywords:** Hepatocellular carcinoma, Histological subtype, Stage, Grade, WHO, Classification, Review, Macrotrabecular massive, Steatohepatitic, Scirrhous

## Abstract

The World Health Organization (WHO) 2019 classification of hepatocellular carcinoma (HCC) introduces new histological subtypes aiding morphomolecular classification. We aimed to reclassify and restage archival HCC following central pathology review and evaluate the frequency of new subtypes in a large European HCC cohort. All histological material of 100 curative liver resection specimens for HCC from 100 patients (80 male, median age 72, IQR: 61.5–76.5 years, BCLC stage 0-C) operated from 2001 to 2018 was centrally reviewed. All cases were reclassified according to WHO 2019 and restaged according to TNM 2017 system. Thirty-five HCCs (35%) were classified into new subtypes: 15 macrotrabecular-massive (15%), 7 steatohepatitic (7%), 4 scirrhous (4%), 3 fibrolamellar (3%), 2 chromophobe (2%), 2 lymphocyte-rich (2%), 1 clear cell (1%), and 1 neutrophil-rich (1%). Histological grade changed in 45% HCCs (*p* < 0.001). TNM stage was modified from 1 to 2 in 22/100 HCCs (*p* < 0.001). The main histological pattern was solid (52%), pseudoglandular (17%), trabecular (16%), and macrotrabecular (15%). Microvascular invasion was detected in 64% HCCs. The non-neoplastic liver parenchyma showed steatosis (40%), steatohepatitis (19%), and/or chronic inflammation (71%). Fibrosis was staged as F0 35%, F1 17%, F2 13%, F3 11%, or F4 24%. Central histopathology review according to WHO 2019 and TNM 2017 modified histological grading in approximately half and staging in one quarter of resected HCCs, respectively, with clinical implications. One-third of HCCs were reclassified into new histological subtypes, some with known molecular background, prognostic and/or predictive impact, important for patient management.

**Supplementary Information:**

The online version contains supplementary material available at 10.1007/s00428-025-04301-4.

## Introduction

Hepatocellular carcinoma (HCC) is the most common type of primary liver cancer, accounting for up to 90% of the cases, and a leading cause of cancer-related mortality globally [1, 2]. Incidence rates have been increasing steadily in many countries, particularly in Northern and Western Europe, Australia, Northern America, and Southern Africa [1]. In Greece, liver cancer is a public health challenge and ranks as the 11th most common cancer, with 1669 new cases in 2022, and the 7th cause of cancer-related mortality [3].

HCC is a typical inflammation-associated cancer and more than 90% develop in the setting of chronic liver disease with necro-inflammation [3]. Major risk factors include chronic hepatitis B virus (HBV) or hepatitis C (HCV) infection, alcohol-related liver disease, and metabolic dysfunction-associated steatotic liver disease (MASLD) related to metabolic syndrome, diabetes, and obesity [4]. Although vaccination and new antiviral treatments decreased HCC cases attributed to viral infections, metabolic dysfunction-associated steatohepatitis (MASH) is the most rapidly growing etiological factor, especially in the West, mainly due to lifestyle habits [5]. Despite noteworthy progress in the development of new therapeutic strategies, HCC prognosis remains devastating with a mean 5-year survival rate of 18% [6].

Mounting evidence supports that HCC is a highly heterogeneous entity, both histologically and genetically, with specific clinicopathologic and molecular features [7]. In this context, in 2019 the 5th edition of World Health Organization (WHO) Classification of Tumors of the Digestive system has introduced new HCC subtypes with unique clinical, morphological, molecular, and histopathologic characteristics comprising up to 35% of cases [8]. These subtypes that are distinct from the conventional not otherwise specified (NOS) histological type include steatohepatitic (SH-HCC) (5–20% of HCC), clear cell (3–7% of HCC), macrotrabecular massive (MTM-HCC) (5%), scirrhous (4%), chromophobe (3%), fibrolamellar, neutrophil-rich, and lymphocyte-rich HCC [8]. Of note, only the scirrhous and fibrolamellar subtype have been officially described in the 4th edition [9]. It is expected that recognition of these new subtypes will offer significant prognostic information that may bridge the gap between tumor biology and therapeutic interventions.

Currently, HCC is histologically staged according to the 8th edition of the American Joint Committee on Cancer (AJCC) [10], which includes notable modifications in the T category compared to the previous edition [11].

HCC new histological subtypes have not been adequately studied and their exact definition based on morphology varies [12]. There are limited data on the frequency of new HCC subtypes, especially in White European population. The purpose of this study was to review liver resection specimens with the diagnosis of “HCC” re-evaluating histological patterns and subtypes and recategorizing them according to the updated WHO 2019 HCC classification system and the 8th AJCC edition.

## Methods

### Patients

This was a retrospective descriptive cross-sectional study of liver resection specimens from anonymized patients with HCC who were treated at the Surgical Departments of Aretaieion and Attikon University Hospitals between 2001 and 2018. The study was approved by the Research Ethics Committee, Medical School, National and Kapodistrian University of Athens (protocol no: 106/13–02–2019, 191/15–03–2019). According to internal hospital policy and the ethical guidelines of the Declaration of Helsinki, all patients had given pre-operatively consent regarding the use of their anonymized data for research purposes [13]. The clinical, demographic, and laboratory data, including age at surgical resection, sex, risk factors of chronic liver disease, and Barcelona Clinic Liver Cancer (BCLC) stage were obtained from the electronic hospital medical records.

### Histological assessment

Anonymized histological reports and all formalin-fixed, paraffin-embedded hematoxylin–eosin slides, slides with special stains, and immunostained slides from each case were retrieved from the Department of Pathology, Aretaieion Hospital and the 2nd Department of Pathology, Attikon University Hospital and were centrally reviewed by an expert hepatopathologist together with a group of general pathologists at a multi-headed microscope. All tissue sections were evaluated and scored blindly to the clinico-pathological information. The cases were reclassified according to the updated 2019 WHO [8] and 8th AJCC edition classification systems [10]. Additional features reviewed were tumor maximum size and number, histological grade/histological differentiation, nuclear grade, growth pattern, microvascular invasion (MVI), and background liver disease.

Histological subtypes, tumor histological grade, and growth patten were evaluated based on the 2019 WHO classification [8]. SH-HCC was characterized by histological features of steatohepatitis in at least 50% of tumor area, including macrovesicular steatosis, ballooning, pericellular fibrosis, and at least mild intratumoral inflammation. Steatohepatitic morphology in < 50% of tumor area was noted in HCC otherwise classified as NOS. Clear cell subtype consisted of clear cell morphology from glycogen accumulation in ≥ 80% of the tumor with some degree of steatosis acceptable within the tumor. MTM-HCC were defined as having trabeculae ≥ 10 cell-thick comprising > 50% of the tumor. Scirrhous subtype required the presence of dense fibrosis with clusters of neoplastic cells in > 50% of the tumor. Chromophobe HCC was characterized by light, almost clear cytoplasm, abrupt focal nuclear anaplasia and pseudocysts. Fibrolamellar HCC was defined as a tumor composed of large, polygonal cells with eosinophilic granular cytoplasm and prominent nucleoli within extensive lamellar stromal fibrosis. Neutrophil-rich subtype was characterized by diffuse intratumoral infiltration by numerous neutrophils. This subtype may show focal or extensive sarcomatoid differentiation. Lymphocyte-rich subtype included HCC with prominent lymphocytic infiltrates outnumbering tumor cells in most fields. Tumors that could not be classified as any of the above subtypes were considered as HCC NOS.

All HCC were re-graded according to the three-tiered WHO 2019 scheme that combines architectural and nuclear features recognizing well, moderately, and poorly differentiated tumors (grade 1, 2, and 3, accordingly) [8]. When more than one grade was present in the tumor, the worst grade was reported and the extent of other grades was recorded. HCC were also categorized by their principal histological growth pattern, i.e., trabecular, solid, pseudoglandular, and macrotrabecular. The predominant pattern was recorded in cases with more than one growth patterns, and the second more frequent pattern was also noted. Nuclear grade was evaluated according to the system proposed in the Armed Forces Institute of Pathology “Tumors of the liver and intrahepatic bile ducts” 2001 edition focusing on the severity of nuclear pleomorphism [14]. In more detail, nuclear grade 1 HCC were undistinguishable from hepatocellular adenomas, grade 2 had prominent nucleoli, hyperchromatism and nuclear membrane irregularity, grade 3 had greater nuclear pleomorphism and angulated nuclei, and grade 4 HCCs were characterized by marked pleomorphism, hyperchromatism and usually anaplastic giant cells [14, 15].

All tumors were restaged according to the 8th AJCC edition [10]. Changes in the T category compared to the previous edition, included sub-division of pT in pT1a for solitary tumors up to 2 cm regardless of the presence of MVI, and in pT1b for tumors > 2 cm without MVI. In the 7th edition, MVI determined whether the tumor was T1 or T2. Additionally, in the 8th edition, pT2 refers to the exact HCC diameter (2–5 cm), while pT4 stage includes pT3b and pT4 of the 7th edition. Stage pT3a of the 7th edition changes to pT3 as a single category without subdivisions in the 8th edition. T category was determined by reviewing tumor number, maximum tumor size, MVI, major branch of portal vein or hepatic vein invasion, and direct invasion of adjacent organs. Lymph node and distant metastasis were recorded for determination of N and M category, respectively.

Evaluation of the non-neoplastic background liver was based on HCC etiology. For viral and autoimmune etiology, grading of necroinflammatory activity was assessed according to Scheuer et al. as none, minimal/portal, mild, moderate, or severe hepatitis [16], and staging according to METAVIR (F0-F4) [17]. For alcohol-related and MASLD etiology, grade of steatosis, grading of necroinflammatory activity, and staging of fibrosis were evaluated according to NASH CRN histological scoring [18].

Overall survival (OS) was defined as the time between date of surgery and either time of death or last follow-up visit. Only patients with known follow-up data were included in survival analysis. Patients who died within 3 months after surgery due to postoperative complications were excluded from survival analysis.

### Statistical analysis

Descriptive statistics were presented as counts and percentages for qualitative data. Quantitative data were described by the mean and standard deviation or median with interquartile range. Groups were compared with the Ficher’s exact test or Pearson’s chi-square test for qualitative data, and the Student’s *t* test for quantitative data. Survival analysis was performed using the Kaplan–Meier method and comparisons were examined using the log-rank test and the Cox proportional hazards model. All tests were two-sided and a *p* value of 0.05 or less was considered statistically significant. Statistical analyses were performed with the use of SPSS 29.0 software (SPSS Inc., Chicago, USA).

## Results

Of the 109 cases enrolled in the study, five were excluded due to inadequate tissue for histological assessment, unavailable histological slides, or missing clinical information. Four cases were excluded following central review due to equivocal histological features suggestive of combined hepatocellular-cholangiocarcinoma. A total of 100 HCC cases were included in the study.

### Patients’ characteristics

Patient demographics, etiology, and BCLC stage of 100 HCC cases are summarized in Table 1.

**Table 1:** Demographics and clinical features of 100 HCC patients

Patients’ characteristics *n* = 100	*n* (%)
Age median (IQR) years	72 (61.5–76.5)
Sex	
Male	80 (80)
Female	20 (20)
Etiology of chronic liver disease	
Viral hepatitis	41 (41)
*HBV*	*31 (31)*
*HCV*	*8 (8)*
*HBV and HCV*	*2 (2)*
Metabolic	28 (28)
Alcohol	2 (2)
Other/Not known*	29 (29)
*Advanced fibrosis/cirrhosis*	*4 (4)*
*No advanced fibrosis/cirrhosis*	*25 (25)*
BCLC stage	
0	4 (4)
A	62 (62)
B	25 (25)
C	9 (9)
D	0 (0)

IQR interquartile range

^*^ Fibrosis staging in this patient subgroup was included to evaluate the presence of cirrhosis as a potential risk factor for HCC, even in the absence of a known chronic liver disease

### Macroscopic features of HCC

Macroscopic pathology features of 100 HCC included in the study are summarized in Table S1.

### Microscopic histological features and reclassification of HCC after central review

Microscopic histological features of HCC, including subtype, histological differentiation (grade), growth pattern, and stage are summarized in Table 2. Following central review, HCCs initially reported between 2001 and 2018 were classified according to WHO 2019. The majority of HCC (65%) were characterized as NOS. The next most common subtypes were MTM-HCC (15%) and SH-HCC followed by SH (7%). There were 4 scirrhous, 3 fibrolamellar, 2 chromophobe, 2 lymphocyte-rich (2%), 1 clear cell, and 1 neutrophil-rich HCC. Supplementary Figs. S1 and S2 show representative images of HCC histological subtypes.

**Table 2:** Histological features of 100 HCC according to WHO 2019 [8]

Histological features *n* = 100	*n* (%)
Histological subtype	
NOS	65 (65)
Macrotrabecular massive	15 (15)
Steatohepatitic	7 (7)
Scirrhous	4 (4)
Fibrolamellar	3 (3)
Chromophobe	2 (2)
Lymphocyte-rich	2 (2)
Clear cell	1 (1)
Neutrophil-rich	1 (1)
Predominant histological pattern	
Solid	52 (52)
Pseudoglandular	17 (17)
Trabecular	16 (16)
Macrotrabecular	15 (15)
Histological differentiation	
Well differentiated	6 (6)
Moderately differentiated	63 (63)
Poorly differentiated	31 (31)
Nuclear grade*	
1	2 (2)
2	19 (19)
3	64 (64)
4	15 (15)
T stage	
1	33 (33)
*1A*	*4 (4)*
*1B*	*29 (29)*
2	38 (38)
3	23 (23)
4	6 (6)
Microvascular invasion	
Yes	64 (64)
No	36 (36)
Lymph node metastasis	
Yes	2 (2)
No	98 (2)
TNM Stage AJCC 8th edition	
I	31 (31)
II	39 (39)
III	26 (26)
IV	4 (4)**
Bile production	
Yes	41 (41)
No	59 (59)
Necrosis (*n* = 100)	
Absent	36 (36)
Focal	21 (21)
Extensive	43 (43)

NOS not otherwise specified; *according to [14]; ** identified following surgery

**Fig. 1:**
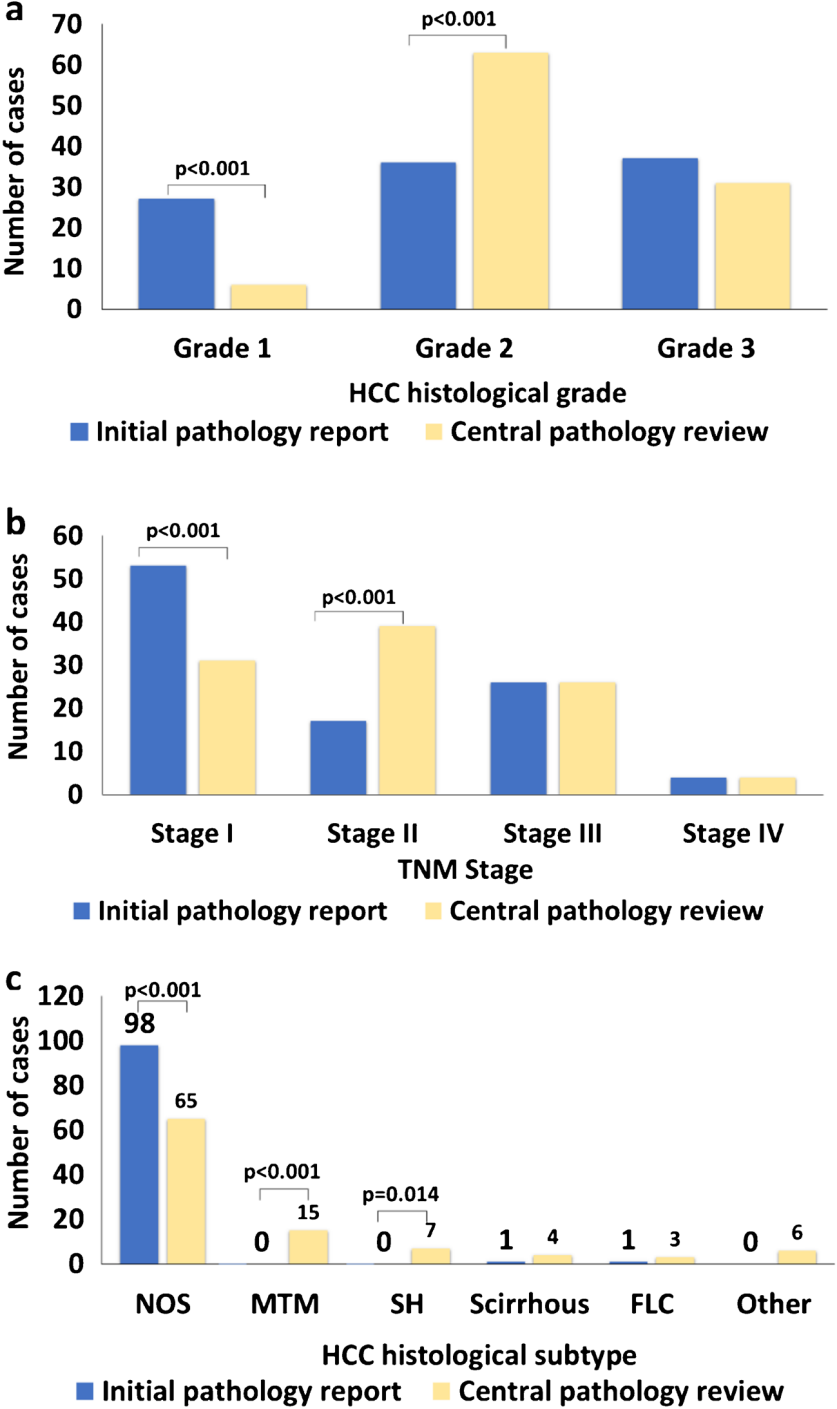
**a** Differences in HCC histological grade following central histopathology review compared to initial pathology report. **b** Differences in HCC TNM stage following central histopathology review compared to initial pathology report **c** Differences in HCC histological subtype following central histopathology review compared to initial pathology report. “Οther” HCC subtypes include chromophobe *n* = 2, lymphocyte-rich *n* = 2, clear cell *n* = 1, and neutrophil-rich *n* = 1. *NOS* not otherwise specified, *MTM* macrotrabecular massive, *SH* steatohepatitic, *FLC* fibrolamellar HCC

Histological features of 100 HCC based on previous WHO and AJCC editions as shown in the initial pathology report and central review results according to the updated WHO 2019 classification and 8th AJCC edition are summarized in Table 3. Thirty-three HCCs (33%) were reclassified into new subtypes compared to initial pathology report (*p* < 0.001) (Fig. 1a). Histological differentiation (grade) changed in 45 (45%) cases and most HCC were revaluated as having worse differentiation with 18 (18%) changing from well to moderate and 6 (6%) changing from moderate to poor (*p* < 0.001) (Table 4, Fig. 1b). TNM stage was modified from I to II in 22/100 HCCs (*p* < 0.001) (Fig. 1c).

**Table 3:** Histopathology findings in the initial pathology report compared to central review of 100 HCC

Initial pathology report	*n* (%)	Central review	*n* (%)	*P* value*
Histological subtype				
*NOS*	98 (98)	*NOS*	65 (65)	** < 0.001**
*Scirrhous*	1 (1)	*Scirrhous*	4 (4)	0.3687
*Fibrolamellar*	1 (1)	*Fibrolamellar*	3 (3)	0.6212
		*Steatohepatitic*	7 (7)	**0.0140**
		*Clear cell*	1 (1)	1.000
		*Lymphocyte-rich*	2 (2)	0.4975
		*Macrotrabecular massive*	15 (15)	** < 0.001**
		*Chromophobe*	2 (2)	0.4975
		*Neutrophil-rich*	1 (1)	1.000
Histological differentiation				
*Well differentiated*	27 (27)	*Well differentiated*	6 (6)	** < 0.001**
*Moderately differentiated*	36 (36)	*Moderately differentiated*	63 (63)	** < 0.001**
*Poorly differentiated*	37 (37)	*Poorly differentiated*	31 (31)	0.4556
TNM stage				
*I*	53 (53)	*I*	31 (31)	** < 0.001**
*II*	17 (17)	*II*	39 (39)	** < 0.001**
*III*	26 (26)	*III*	26 (26)	1.000
*IV*	4 (4)	*IV*	4 (4)	1.000

^*^Numbers in bold denote statistically significant p values

**Table 4:** Reclassification status of 100 HCC following central histopathology review

Reclassification status *n* = 100	*n* (%)
Histological subtype	
Not reclassified	67 (67)
*NOS*	65 (65)
*Fibrolamellar*	1 (1)
*Scirrhous*	1 (1)
Reclassified	33 (33)
*NOS to macrotrabecular massive*	15 (15)
*NOS to steatohepatitic*	7 (7)
*NOS to scirrhous*	3 (3)
*NOS to fibrolamellar*	2 (2)
*NOS to chromophobe*	2 (2)
*NOS to lymphocyte-rich*	2 (2)
*NOS to neutrophil-rich*	1 (1)
*NOS to clear cell*	1 (1)
Histological differentiation	
Not reclassified	55 (55)
*Well differentiated*	5 (5)
*Moderately differentiated*	29 (29)
*Poorly differentiated*	21 (21)
Reclassified	45 (45)
*Well to moderate*	18 (18)
*Well to poor*	4 (4)
*Moderate to well*	1 (1)
*Moderate to poor*	6 (6)
*Poor to moderate*	16 (16)
TNM Stage AJCC 8th edition	
Not changed	78 (78)
*I*	31 (31)
*II*	17 (17)
*III*	26 (26)
*IV*	4 (4)
Changed *I to II*	22 (22)

### Background liver histology status of HCCs

Background non-neoplastic liver histological features are shown in Table S2. Histological examination revealed steatosis in 40%, steatohepatitis in 19%, and/or chronic inflammation in 71% of cases. Fibrosis was absent in 35% (F0), while 17%, 13%, 11%, and 24% of the cases were classified as F1, F2, F3, and F4, respectively.

### Survival analysis

The mean duration of follow-up was 54 months (range 1–197 months). Patients with HCC of higher histological grade and nuclear grade had worse prognosis (*p* = 0.034 and *p* = 0.049, respectively). Moreover, multifocality, presence of MVI, margin invasion, portal vein thrombosis, advanced TNM, and advanced BCLC stage were significantly associated with lower overall survival (*p* = 0.029, *p* = 0.007, *p* = 0.032, *p* = 0.007, *p* = 0.013, and *p* = 0.012, respectively). Table S3 shows patient OS according to histological HCC subtypes. HCC histological subtypes did not show significant correlation with OS (*p* = 0.162).

## Discussion

To our knowledge, this is the first study to evaluate the impact of the reclassification of HCC subtype, histological differentiation and stage according to the WHO 2019 classification of HCC and AJCC staging system 8th edition following central histopathology review. Approximately one-third of HCCs were reclassified into new histological subtypes. In the initial pathology reporting, only two subtypes were recognized with one case each, fibrolamellar and scirrhous, since these were the only HCC subtypes described in the WHO 4th edition [9]. On reclassification, among the cases that were assigned to distinct subtypes, MTM-HCC and SH-HCC were the most common, followed by other less frequent subtypes such us scirrhous, fibrolamellar, chromophobe, clear cell, neutrophil-rich, and lymphocyte-rich. The percentage of HCCs that were classified into new subtypes (35%) is similar to that reported in the WHO 2019 classification. Our results regarding subtype frequency are similar to those described in the 5th WHO edition and in two large HCC cohorts with the exception of MTM-HCC that was more common in our study (15% vs. 3.9% and 9%) [8, 19, 20]. Other studies focusing on MTM-HCC histopathology also describe increased frequency of MTM subtype in HCC cohorts [21–24].

HCC subtyping, based on the WHO 2019 classification, is advantageous as it integrates the particular clinical, pathological, genetic, epigenetic, and immunophenotypic features of each subtype [8]. SH-HCCs are usually well differentiated tumors that frequently occur in patients with one or more known risk factors for MASLD but it has also been reported in cases without background liver disease [12, 19, 25]. In a recent large HCC cohort, Trapani et al. demonstrated the association between SH-HCC and metabolic syndrome as well as with MASLD, while 38% of patients had no steatosis in the non-tumoral liver [12]. In our study, 5 out of 7 patients with SH-HCC had risk factors for MASLD, one had steatohepatitis, and two had steatotic background liver. MTM HCCs arise more frequently in noncirrhotic patients and are reportedly associated with HBV infection and high serum alpha-fetoprotein (AFP) levels [26]. Several studies have reported that MTM HCC are often larger, higher stage and poorly differentiated with vascular invasion [19, 23, 27]. Similarly, in our study, 7 out of 15 patients with MTM-HCC had HBV infection, 11 presented MVI, 9 had large tumor size (> 9 cm), and 14 were moderately or poorly differentiated. On the other hand, clear cell and scirrhous HCCs are mostly well to moderately differentiated [8, 28]. Neutrophil-rich variant is characterized by elevated white blood cell count, C-reactive protein and IL-6 and the tumor produces G-CSF, while lymphocyte-rich HCC has frequently programmed death-ligand 1 (PD-L1) expression [8, 29, 30]. Compared to conventional HCC NOS, clear cell and lymphocyte-rich subtypes have favorable prognosis, MTM- and neutrophil-rich have worse prognosis, while SH-, chromophobe, and fibrolamellar HCC have similar prognosis [8, 12, 19, 20, 23, 24, 26, 29–33]. In our study, no statistical differences were found in OS between HCC subtypes and HCC NOS. This discrepancy may be due to the small case numbers for each subtype precluding accurate statistical analysis.

Three tumors previously categorized as NOS were reclassified as scirrhous and two as fibrolamellar HCC, although both subtypes were described in the previous WHO classification. This may be attributed to the fact that the updated WHO criteria provide clearly defined morphological features for these subtypes. Moreover, the initial histological evaluation was not performed by an expert hepatopathologist; therefore, some histological features may have been under-recognized.

Recent guidelines on HCC management recommend tumor biopsy prior to pharmaceutical treatment in order to obtain relevant prognostic, including tumor differentiation and histological subtyping, and possibly predictive information [34, 35]. In this context, the clinical relevance of HCC subtyping has increased since there is an established correlation between morphological, molecular, clinical, and histological features of HCC and a possible predictive value of some molecular changes, such as *CTTNB1* mutation [36]. Calderaro et al. introduced a morphomolecular HCC classification based on tumor proliferative activity and showed that the histological growth patterns and subtypes are associated with specific molecular alterations [37]. The high proliferative subgroup (molecular classes G1–G3) includes HCCs characterized by TP53 mutation, vascular invasion, poor differentiation, high AFP levels, HBV infection clear cell or sarcomatoid features, and compact or MTM growth pattern. The low proliferative subgroup (G4–G6 molecular classes) includes HCCs that show *CTNNB1* mutation (except for G4 class), are well differentiated, are related to HCV infection, and are usually of small size, with trabecular or pseudoglandular pattern, without satellite nodules or vascular invasion [37]. Treatment decisions in HCC are challenging involving multimodal approaches, mainly due to tumor molecular and immunological heterogeneity. Moreover, HCC patients require treatment of both cancer and the underlying chronic liver disease [38]. For over a decade, tyrosine kinase inhibitors were the only option but recently, immune-checkpoint inhibitor (ICI) or ICI/anti-VEGF combinations are incorporated in first-line treatment [34, 39]. Assigning each HCC case to an appropriate morphomolecular subtype can add novel therapeutic options and improve patient care.

Central histopathology review modified histological differentiation and TNM staging in approximately 1/2 and 1/4 of resected HCCs, respectively. Most cases were reclassified into worse grading, providing important prognostic information that could bridge the gap between clinico-pathological parameters and patient outcome. It is widely accepted that tumor grade predicts OS and recurrence-free survival after resection, as high grade HCCs are more aggressive, more invasive and tend to metastasize more frequently [15]. Thirty-four cases were reclassified from well or poorly differentiated to moderately differentiated tumors. This shift may reflect the standardized approach of the WHO 2019 HCC grading system that is based on global assessment of morphology and on well-defined cellular features [8]. In addition, the central review performed by an expert hepatopathologist improved consistency. Therefore, the difference noted could imply better reproducibility rather than reduced discriminatory power. TNM stage was modified from I to II in 22% of HCCs according to the 8th AJCC edition. This difference can be attributed to increased detection of MVI compared to the initial pathology report. MVI is a negative prognostic factor for HCC and is associated with advanced tumor stage, distant metastasis, and worse patient survival [21, 40]. However, MVI does not affect long-term survival of patients with solitary HCC ≤ 2 cm [41].

While current clinical practice guidelines for the management of HCC patients propose the BCLC staging system to guide treatment decisions, histological subtypes could provide valuable prognostic information moving toward more personalized approaches [35, 36]. Patients with aggressive HCC subtypes, such as MTM and neutrophil-rich, may be candidates for adjuvant therapy following resection, but not ideal for liver transplantation due to high recurrence risk [23, 26, 31, 42]. However, MTM-HCCs, which are characterized by increased neoangiogenesis, and lymphocyte-rich HCCs could respond to anti-angiogenic agents and ICIs, respectively [23, 26, 29, 31, 43]. SH-HCCs may potentially respond to JAK-STAT inhibitors [43]. Upstaging from T1 to T2 provides additional prognostic information, identifying patients who could benefit from adjuvant therapy [27].

The inclusion of BCLC stage B or C patients in our surgical cohort reflects real-world clinical practice at our center, particularly during earlier years when systemic treatment options were limited. Although the BCLC algorithm generally recommends non-surgical therapies for these stages, selected patients with preserved liver function, limited tumor burden, no portal hypertension, or acceptable performance status may be considered for resection based on multidisciplinary evaluation [44]. This approach aligns with reports showing that, in chosen cases, surgical treatment may offer benefit even in intermediate or advanced BCLC stages [45, 46].

Intratumoral heterogeneity remains a major challenge in the accurate histological characterization of HCC, particularly when relying on limited biopsy material. In our cohort, 34% of tumors exhibited more than one histological growth pattern, highlighting the risk of sampling error. Few studies have investigated the concordance of histopathological characteristics between preoperative needle liver biopsy (NLB) and surgical specimens in HCC [12, 47–50]. Regarding three-tiered histological tumor grade, NLB appears to have good to excellent accuracy ranging from 62.1% [49] to 91.4% [47], but results may vary based on tumor size and number of needle passes. Existing data on histological subtyping based on WHO 2019 HCC classification is very scarce. Trapani et al. [47] have shown diagnostic agreement between NLB and surgical specimen in 7 out of 10 (70%) cases with SH-HCC or HCC NOS with an SH component < 50%, while Lin et al. reported that a subset of 6 cases of MTM-HCC with recurrent lesions diagnosed by NLB had similar histopathological characteristics to the corresponding primary surgical specimen [50].

One of our study’s strengths is the relatively high number of White European cases and according to the best of our knowledge ours is the largest clinico-pathological HCC database in Greece. In addition, our study incorporating the WHO 2019 classification criteria highlights HCC histological heterogeneity and emphasizes changes in the role of pathology in HCC from mere diagnostic to prognostic and, hopefully in the near future, predictive of targeted treatment response. Another strength is the inclusion of resection specimens only since tumor biopsy may be hampered by sampling variability. HCC is histologically heterogeneous with 20–48% including different grades in the same tumor reducing the reliability of HCC grading in needle biopsies [43]. The limitations of our study include its retrospective, non-randomized design, and the challenge of HCC subtyping, as some tumors may include areas fitting different subtypes. Clonal progression, defined by the presence of a morphologically different tumor nodule compared to the rest of the tumor, may prevent accurate tumor subtyping [43]. Our cohort includes only resected HCC, which may introduce selection bias toward patients with better liver function and less advanced fibrosis. This is a known limitation of surgical HCC cohorts. However, the distribution of HCC histological subtypes in our study is consistent with previously published international data and those of WHO 2019 HCC classification, suggesting that our findings may be representative of broader subtype patterns in the European HCC population [8, 19, 20]. Nevertheless, generalizability of our findings may be limited by the single-country cohort, which may restrict applicability of the results to other European or global populations. In addition, the impact of HCC subtype in recurrence-free survival could not be assessed as this information was lacking for most patients in our database due to the absence of an organized nationwide cancer registry.

Central histopathology review of HCC according to WHO 2019 5th edition and AJCC 2017 8th edition modified histological grading, subtyping and staging in a high number of cases. A reproducible and clinically relevant subtyping approach is at the forefront of providing individualized and effective care for HCC patients. Further prospective studies including clinico-pathological, molecular, survival and treatment response data are required to validate our results.

## Supplementary Information

Below is the link to the electronic supplementary material.

Supplementary file1 (DOCX 3.89 MB)

## Data Availability

The data that support the findings of this study are available from the corresponding author upon reasonable request.

## References

[1] Bray F, Laversanne M, Sung H, Ferlay J, Siegel RL, Soerjomataram I, Jemal A (2024) Global cancer statistics 2022: GLOBOCAN estimates of incidence and mortality worldwide for 36 cancers in 185 countries. CA Cancer J Clin 74(3):229–263. 10.3322/caac.21834 38572751 10.3322/caac.21834

[2] Huang DQ, Singal AG, Kanwal F, Lampertico P, Buti M, Sirlin CB, Nguyen MH, Loomba R (2023) Hepatocellular carcinoma surveillance - utilization, barriers and the impact of changing aetiology. Nat Rev Gastroenterol Hepatol 20(12):797–809. 10.1038/s41575-023-00818-8 37537332 10.1038/s41575-023-00818-8

[3] International Agency for Research on Cancer WHO (2024) Cancer fact sheets. https://gco.iarc.fr/today/fact-sheets-cancers. Accessed August 27, 2024

[4] Llovet JM, Kelley RK, Villanueva A, Singal AG, Pikarsky E, Roayaie S, Lencioni R, Koike K, Zucman-Rossi J, Finn RS (2021) Hepatocellular carcinoma. Nat Rev Dis Primers 7(1):6. 10.1038/s41572-020-00240-3 33479224 10.1038/s41572-020-00240-3 PMC13537433

[5] Estes C, Razavi H, Loomba R, Younossi Z, Sanyal AJ (2018) Modeling the epidemic of nonalcoholic fatty liver disease demonstrates an exponential increase in burden of disease. Hepatology 67(1):123–133. 10.1002/hep.29466 28802062 10.1002/hep.29466 PMC5767767

[6] Pessino G, Scotti C, Maggi M, Immuno-Hub C (2024) Hepatocellular carcinoma: old and emerging therapeutic targets. Cancers (Basel). 10.3390/cancers16050901 38473265 10.3390/cancers16050901 PMC10931414

[7] Li X, Ramadori P, Pfister D, Seehawer M, Zender L, Heikenwalder M (2021) The immunological and metabolic landscape in primary and metastatic liver cancer. Nat Rev Cancer 21(9):541–557. 10.1038/s41568-021-00383-9 34326518 10.1038/s41568-021-00383-9

[8] Torbenson MS NI, Park YN, Roncalli M, Sakamato M (2019) Hepatocellular carcinoma. In: WHO Classification of tumours: digestive system tumours. 5th edn. International Agency for Research on Cancer, France, pp 229–239.

[9] Theise ND CMP, Franceschi S, Hytiroglou P, Kudo M (2010) Hepatocellular carcinoma. In: WHO Classification of tumours: digestive system tumours. 4th edn. International Agency for Research on Cancer, France, pp 205–224

[10] Armin MBES, Greene F, Byrd DR, Brookland RK, Washington MK, Gershenwald JE, Compton CC, Hess KR, Sullivan DC, Jessup JM, Brierley JD, Gaspar LE, Schilsky RL, Balch CM, Winchester DP, Asare EA, Madera M, Gress DM, Meyer LR (2017) AJCC cancer staging manual, 8th edn. Springer, New York

[11] Edge SBBD, Compton CC, Fritz AG, Greene FL, Trotti A (2010) AJCC cancer staging manual, 7th edn. Springer, New York

[12] Trapani L, Beaufrere A, Hobeika C, Codjia T, Albuquerque M, Bouattour M, Lesurtel M, Cauchy F, Paradis V (2023) Pathological overview of steatohepatitic hepatocellular carcinoma in a surgical series. Histopathology 83(4):526–537. 10.1111/his.14941 37222200 10.1111/his.14941

[13] Morris K (2013) Revising the declaration of Helsinki. Lancet 381(9881):1889–1890. 10.1016/s0140-6736(13)60951-4 23734387 10.1016/s0140-6736(13)60951-4

[14] Ishak KG, Stocker JT (2001) Tumors of the liver and intrahepatic bile ducts. In: Armed Forces Institute of Pathology. 3rd vol. Washington, D.C., pp 219–220

[15] Martins-Filho SN, Paiva C, Azevedo RS, Alves VAF (2017) Histological grading of hepatocellular carcinoma - a systematic review of literature. Front Med 4:193. 10.3389/fmed.2017.00193 10.3389/fmed.2017.00193 PMC5701623 29209611

[16] Scheuer PJ (1991) Classification of chronic viral hepatitis: a need for reassessment. J Hepatol 13(3):372–374. 10.1016/0168-8278(91)90084-o 1808228 10.1016/0168-8278(91)90084-o

[17] Bedossa P, Poynard T (1996) An algorithm for the grading of activity in chronic hepatitis C. the METAVIR cooperative study group. Hepatology 24(2):289–293 8690394 10.1002/hep.510240201

[18] Kleiner DE, Brunt EM, Van Natta M, Behling C, Contos MJ, Cummings OW, Ferrell LD, Liu YC, Torbenson MS, Unalp-Arida A, Yeh M, McCullough AJ, Sanyal AJ, Nonalcoholic Steatohepatitis Clinical Research N (2005) Design and validation of a histological scoring system for nonalcoholic fatty liver disease. Hepatology 41(6):1313–1321. 10.1002/hep.20701 15915461 10.1002/hep.20701

[19] Shin SH, Park JY, Hwang C, Lee HJ, Shin DH, Kim JY, Ryu JH, Yang KH, Lee TB, Lee JH (2023) Histological subtypes of hepatocellular carcinoma: their clinical and prognostic significance. Ann Diagn Pathol 64:152134. 10.1016/j.anndiagpath.2023.152134 37004359 10.1016/j.anndiagpath.2023.152134

[20] Sweed D, Sweed E, Moaz I, Mosbeh A, Fayed Y, Elhamed SMA, Sweed E, Macshut M, Abdelsattar S, Kilany S, Saied SA, Badr R, Abdallah MS, Ehsan N (2022) The clinicopathological and prognostic factors of hepatocellular carcinoma: a 10-year tertiary center experience in Egypt. World J Surg Oncol 20(1):298. 10.1186/s12957-022-02764-2 36117166 10.1186/s12957-022-02764-2 PMC9484175

[21] Lauwers GY, Terris B, Balis UJ, Batts KP, Regimbeau JM, Chang Y, Graeme-Cook F, Yamabe H, Ikai I, Cleary KR, Fujita S, Flejou JF, Zukerberg LR, Nagorney DM, Belghiti J, Yamaoka Y, Vauthey JN, International Cooperative Study Group on Hepatocellular C (2002) Prognostic histologic indicators of curatively resected hepatocellular carcinomas: a multi-institutional analysis of 425 patients with definition of a histologic prognostic index. Am J Surg Pathol 26(1):25–34. 10.1097/00000478-200201000-00003 11756766 10.1097/00000478-200201000-00003

[22] Rastogi A, Maiwall R, Ramakrishna G, Modi S, Taneja K, Bihari C, Kumar G, Patil N, Thapar S, Choudhury AK, Mukund A, Pamecha V, Sarin SK (2021) Hepatocellular carcinoma: clinicopathologic associations amidst marked phenotypic heterogeneity. Pathol Res Pract 217:153290. 10.1016/j.prp.2020.153290 33307344 10.1016/j.prp.2020.153290

[23] Ziol M, Pote N, Amaddeo G, Laurent A, Nault JC, Oberti F, Costentin C, Michalak S, Bouattour M, Francoz C, Pageaux GP, Ramos J, Decaens T, Luciani A, Guiu B, Vilgrain V, Aube C, Derman J, Charpy C, Zucman-Rossi J, Barget N, Seror O, Ganne-Carrie N, Paradis V, Calderaro J (2018) Macrotrabecularmassive hepatocellular carcinoma: a distinctive histological subtype with clinical relevance. Hepatology 68(1):103–112. 10.1002/hep.29762 29281854 10.1002/hep.29762

[24] Shan Y, Yu X, Yang Y, Sun J, Wu S, Mao S, Lu C (2022) Nomogram for the preoperative prediction of the macrotrabecular-massive subtype of hepatocellular carcinoma. J Hepatocell Carcinoma 9:717–728. 10.2147/JHC.S373960 35974953 10.2147/JHC.S373960 PMC9375985

[25] Yeh MM, Liu Y, Torbenson M (2015) Steatohepatitic variant of hepatocellular carcinoma in the absence of metabolic syndrome or background steatosis: a clinical, pathological, and genetic study. Hum Pathol 46(11):1769–1775. 10.1016/j.humpath.2015.07.018 26410018 10.1016/j.humpath.2015.07.018

[26] Sessa A, Mule S, Brustia R, Regnault H, Galletto Pregliasco A, Rhaiem R, Leroy V, Sommacale D, Luciani A, Calderaro J, Amaddeo G (2022) Macrotrabecular-massive hepatocellular carcinoma: light and shadow in current knowledge. J Hepatocell Carcinoma 9:661–670. 10.2147/JHC.S364703 35923611 10.2147/JHC.S364703 PMC9342198

[27] Fuster-Anglada C, Mauro E, Ferrer-Fabrega J, Caballol B, Sanduzzi-Zamparelli M, Bruix J, Fuster J, Reig M, Diaz A, Forner A (2024) Histological predictors of aggressive recurrence of hepatocellular carcinoma after liver resection. J Hepatol 81(6):995–1004. 10.1016/j.jhep.2024.06.018 38925272 10.1016/j.jhep.2024.06.018

[28] Franses JW, Duda DG (2024) Scirrhous HCC: another ’omic thread in the HCC tapestry. Hepatology 79(4):747–748. 10.1097/HEP.0000000000000609 37725712 10.1097/HEP.0000000000000609

[29] Tsutsui K, Nakayama M, Ogasawara S, Akiba J, Kondo R, Mihara Y, Yano Y, Mizuochi S, Kinjo Y, Murotani K, Yano H (2023) Clinicopathological characteristics and molecular analysis of lymphocyte-rich hepatocellular carcinoma. Hum Pathol 141:43–53. 10.1016/j.humpath.2023.09.004 37742944 10.1016/j.humpath.2023.09.004

[30] Vij M, Veerankutty FH, Raju LP, Gowrishankar G, Rajalingam R, Jothimani D, Kaliamoorthy I, Rammohan A, Rela M (2023) Frequent expression of PD-L1 in lymphocyte-rich hepatocellular carcinoma: a report of 4 cases. Ann Diagn Pathol 66:152172. 10.1016/j.anndiagpath.2023.152172 37348413 10.1016/j.anndiagpath.2023.152172

[31] Jeon Y, Benedict M, Taddei T, Jain D, Zhang X (2019) Macrotrabecular hepatocellular carcinoma: an aggressive subtype of hepatocellular carcinoma. Am J Surg Pathol 43(7):943–948. 10.1097/PAS.0000000000001289 31135484 10.1097/PAS.0000000000001289

[32] Kang HJ, Oh JH, Kim YW, Kim W, An J, Sung CO, Kim J, Shim JH, Hwang S, Yu E, Heaphy CM, Hong SM (2021) Clinicopathological and molecular characterization of chromophobe hepatocellular carcinoma. Liver Int 41(10):2499–2510. 10.1111/liv.14975 34036718 10.1111/liv.14975

[33] Salomao M, Remotti H, Vaughan R, Siegel AB, Lefkowitch JH, Moreira RK (2012) The steatohepatitic variant of hepatocellular carcinoma and its association with underlying steatohepatitis. Hum Pathol 43(5):737–746. 10.1016/j.humpath.2011.07.005 22018903 10.1016/j.humpath.2011.07.005

[34] Suddle A, Reeves H, Hubner R, Marshall A, Rowe I, Tiniakos D, Hubscher S, Callaway M, Sharma D, See TC, Hawkins M, Ford-Dunn S, Selemani S, Meyer T (2024) British Society of Gastroenterology guidelines for the management of hepatocellular carcinoma in adults. Gut 73(8):1235–1268. 10.1136/gutjnl-2023-331695 38627031 10.1136/gutjnl-2023-331695 PMC11287576

[35] European Association for the Study of the Liver. Electronic address eee, European Association for the Study of the L (2024) EASL Clinical Practice Guidelines on the management of hepatocellular carcinoma. J Hepatol. 10.1016/j.jhep.2024.08.028 10.1016/j.jhep.2024.08.028 39690085

[36] Choi JH, Thung SN (2023) Advances in histological and molecular classification of hepatocellular carcinoma. Biomedicines 11(9):2582. 10.3390/biomedicines11092582 37761023 10.3390/biomedicines11092582 PMC10526317

[37] Calderaro J, Couchy G, Imbeaud S, Amaddeo G, Letouze E, Blanc JF, Laurent C, Hajji Y, Azoulay D, Bioulac-Sage P, Nault JC, Zucman-Rossi J (2017) Histological subtypes of hepatocellular carcinoma are related to gene mutations and molecular tumour classification. J Hepatol 67(4):727–738. 10.1016/j.jhep.2017.05.014 28532995 10.1016/j.jhep.2017.05.014

[38] Kinsey E, Lee HM (2024) Management of hepatocellular carcinoma in 2024: the multidisciplinary paradigm in an evolving treatment landscape. Cancers (Basel) 16(3):666. 10.3390/cancers16030666 38339417 10.3390/cancers16030666 PMC10854554

[39] Fortuny M, Sanduzzi-Zamparelli M, Reig M (2024) Systemic therapies in hepatocellular carcinoma: a revolution? United Eur Gastroenterol J 12(2):252–260. 10.1002/ueg2.12510 10.1002/ueg2.12510 PMC10954433 38267015

[40] Rodriguez-Peralvarez M, Luong TV, Andreana L, Meyer T, Dhillon AP, Burroughs AK (2013) A systematic review of microvascular invasion in hepatocellular carcinoma: diagnostic and prognostic variability. Ann Surg Oncol 20(1):325–339. 10.1245/s10434-012-2513-1 23149850 10.1245/s10434-012-2513-1

[41] Shindoh J, Andreou A, Aloia TA, Zimmitti G, Lauwers GY, Laurent A, Nagorney DM, Belghiti J, Cherqui D, Poon RT, Kokudo N, Vauthey JN (2013) Microvascular invasion does not predict long-term survival in hepatocellular carcinoma up to 2 cm: reappraisal of the staging system for solitary tumors. Ann Surg Oncol 20(4):1223–1229. 10.1245/s10434-012-2739-y 23179993 10.1245/s10434-012-2739-y PMC3856190

[42] Vij M, Raju LP, Jothimani D, Subbiah K, Simon E, Gowrishankar G, Rajalingam R, Kaliamoorthy I, Rammohan A, Rela M (2025) Clinicopathological characteristics of neutrophil-rich hepatocellular carcinoma: an uncommon subtype of primary liver cancer. Int J Surg Pathol 33(4):828–841. 10.1177/10668969241291882 39533751 10.1177/10668969241291882

[43] Torbenson MS (2021) Hepatocellular carcinoma: making sense of morphological heterogeneity, growth patterns, and subtypes. Hum Pathol 112:86–101. 10.1016/j.humpath.2020.12.009 33387587 10.1016/j.humpath.2020.12.009 PMC9258523

[44] Reig M, Forner A, Rimola J, Ferrer-Fabrega J, Burrel M, Garcia-Criado A, Kelley RK, Galle PR, Mazzaferro V, Salem R, Sangro B, Singal AG, Vogel A, Fuster J, Ayuso C, Bruix J (2022) BCLC strategy for prognosis prediction and treatment recommendation: the 2022 update. J Hepatol 76(3):681–693. 10.1016/j.jhep.2021.11.018 34801630 10.1016/j.jhep.2021.11.018 PMC8866082

[45] Lopez-Lopez V, Kalt F, Zhong JH, Guidetti C, Magistri P, Di Benedetto F, Weinmann A, Mittler J, Lang H, Sharma R, Vithayathil M, Tariq S, Sanchez-Velazquez P, Rompianesi G, Troisi RI, Gomez-Gavara C, Dalmau M, Sanchez-Romero FJ, Llamoza C, Tschuor C, Deniz U, Lurje G, Husen P, Hugli S, Jonas JP, Rossler F, Kron P, Ramser M, Ramirez P, Lehmann K, Robles-Campos R, Eshmuminov D (2024) The role of resection in hepatocellular carcinoma BCLC stage B: a multi-institutional patient-level meta-analysis and systematic review. Langenbecks Arch Surg 409(1):277. 10.1007/s00423-024-03466-x 39269544 10.1007/s00423-024-03466-x PMC11399194

[46] Pandrowala S, Patkar S, Goel M, Mirza D, Mathur SK (2023) Surgical resection for large hepatocellular carcinoma and those beyond BCLC: systematic review with proposed management algorithm. Langenbecks Arch Surg 408(1):144. 10.1007/s00423-023-02881-w 37041364 10.1007/s00423-023-02881-w

[47] Colecchia A, Scaioli E, Montrone L, Vestito A, Di Biase AR, Pieri M, D’Errico-Grigioni A, Bacchi-Reggiani ML, Ravaioli M, Grazi GL, Festi D (2011) Pre-operative liver biopsy in cirrhotic patients with early hepatocellular carcinoma represents a safe and accurate diagnostic tool for tumour grading assessment. J Hepatol 54(2):300–305. 10.1016/j.jhep.2010.06.037 21056498 10.1016/j.jhep.2010.06.037

[48] Wu JS, Feng JL, Zhu RD, Liu SG, Zhao DW, Li N (2019) Histopathological characteristics of needle core biopsy and surgical specimens from patients with solitary hepatocellular carcinoma or intrahepatic cholangiocarcinoma. World J Gastrointest Oncol 11(5):404–415. 10.4251/wjgo.v11.i5.404 31139310 10.4251/wjgo.v11.i5.404 PMC6522762

[49] Wang L, Wang J, Zhang X, Li J, Wei X, Cheng J, Ling Q, Xie H, Zhou L, Xu X, Zheng S (2015) Diagnostic value of preoperative needle biopsy for tumor grading assessment in hepatocellular carcinoma. PLoS One 10(12):e0144216. 10.1371/journal.pone.0144216 26658912 10.1371/journal.pone.0144216 PMC4682812

[50] Lin C, He Y, Liu M, Wu A, Zhang J, Li S, Cao Q, Liu F (2023) Vessels that encapsulate tumor clusters (VETC) predict cTACE response in hepatocellular carcinoma. J Hepatocell Carcinoma 10:383–397. 10.2147/JHC.S395903 36915392 10.2147/JHC.S395903 PMC10007987

