# Supplementary material for: Central histopathological review of a European hepatocellular carcinoma cohort: impact of the WHO 2019 classification on histological diagnosis and TNM staging
Source: Virchows Arch. 2025 Oct 24;488(3):639–49. doi: 10.1007/s00428-025-04301-4 (PMC12963078; doi:10.1007/s00428-025-04301-4)
Supplement: Supplementary file 1 — Supplementary file1 (DOCX 3.89 MB) [file 428_2025_4301_MOESM1_ESM.docx]

**SUPPLEMENTARY MATERIAL**

**Central histopathological review of a European hepatocellular carcinoma cohort: impact of the WHO 2019 classification on histological diagnosis and TNM staging**

Konstantina Dimopoulou^1^, Despoina Myoteri^2^, John Contis^3^, Panagis Lykoudis^4^, Constantinos Nastos^4^, Georgios Fragulidis^3^, Antonios Vezakis^3^, Dionysios Dellaportas^5^, Manousos Konstadoulakis^3^, Ioannis G Panayiotides^6^, Nikolaos Arkadopoulos^5^, Periklis G. Foukas^6*^, Dina Tiniakos^2,7*^

*Joint senior authors

**Supplementary** **Table S1.** Macroscopic pathology features of 100 HCC

| **Macroscopic features**  n=100 | **n (%)** |
| --- | --- |
| **Tumour greatest diameter** |  |
| ≤ 2 cm | 5 (5) |
| 2-5 cm | 24 (24) |
| 5-10 cm | 47 (47) |
| >10 cm | 24 (24) |
| **Number of nodules** |  |
| single | 68 (68) |
| multiple | 32 (32) |
| *2-3* | *13 (13)* |
| *>3* | *19 (19)* |
| **Satellite nodules** |  |
| Yes | 24 (24) |
| No | 76 (76) |
|  |  |
| **Macrovascular invasion** |  |
| Yes | 2 (2) |
| No | 98 (98) |
| **Margin invasion** |  |
| Yes | 15 (15) |
| No | 83 (83) |
| Not recorded | 2 (2) |
| **Liver capsule invasion** |  |
| Yes | 15 (15) |
| No | 58 (58) |
| Not recorded | 27 (27) |
| **Liver capsule rupture** |  |
| Yes | 4 (4) |
| No | 69 (69) |
| Not recorded | 27 (27) |

**Supplementary Table S2.** Background liver histological features of 100 HCC cases

| **Background liver histology**  n=100 | **n (%)** |
| --- | --- |
| Chronic hepatitis | 55 (55) |
| Activity grade*^,^ ** |  |
| *Absent* | 23 (30) |
| *Minimal/Mild* | 46 (59) |
| *Moderate* | 9 (11) |
| *Severe* | 1. (0) |
| Steatohepatitis** | 19 (19) |
| Steatosis grade** |  |
| *S0* | 60 (60) |
| *S1* | 30 (30) |
| *S2* | 10 (10) |
| *S3* | 1. (0) |
| Fibrosis stage*^,^** |  |
| *F0* | 35 (35) |
| *F1* | 17 (17) |
| *F2* | 13 (13) |
| *F3* | 11 (11) |
| *F4* | 24 (24) |

*Histological evaluation according to *[[18](#_ENREF_18)] or **[[16](#_ENREF_16)] based on individual case aetiology*

**Supplementary Table S3.** Overall survival of HCC patients according to histological subtype

| **HCC histological subtypes** | **n*** | **Mean OS (months) ± SD (range)** |
| --- | --- | --- |
| NOS | 37 | 63.2 ± 51 (4-197) |
| Macrotrabecular massive | 8 | 46.3 ± 41.8 (6-138) |
| Steatohepatitic | 5 | 50 ± 19.5 (50±19.5) |
| Scirrhous | 2 | 55.5 ± 33.2 (32-79) |
| Fibrolamellar | 2 | 130.5 ± 5 (127-134) |
| Chromophobe | 2 | 97.5 ± 112.4 (18-177) |
| Lymphocyte-rich | 2 | 36 ± 19.8 (22-50) |
| Neutrophil-rich | 0 | N/A |
| Clear cell | 0 | N/A |

N/A not applicable, OS overall survival

*Only patients with known follow-up data (n=58) were included in survival analysis

**Supplementary Figure Legends**

**Supplementary Figure S1**. **a.** Steatohepatitic HCC, haematoxylin-eosin (H-E) x200; **b.** Clear cell HCC, H-E, x200; **c.** Macrotrabecular massive HCC, H-E x200; **d.** Scirrhous HCC, H-E x100.

**Supplementary Figure S2**. **a.** Chromophobe HCC, haematoxylin-eosin (H-E) x50. Inset shows abrupt anaplasia, H-E x400; **b.** Fibrolamellar HCC, H-E, x200; **c.** Neutrophil-rich HCC, H-E x400; **d.** Lymphocyte-rich HCC, H-E x200.


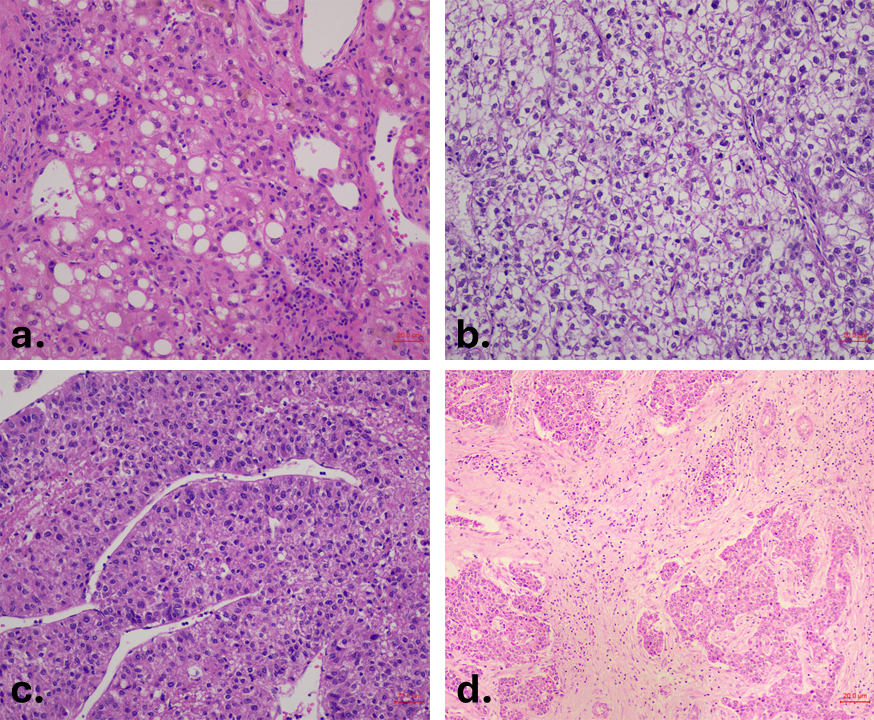


**Supplementary Figure S1**. **a.** Steatohepatitic HCC, haematoxylin-eosin (H-E) x200; **b.** Clear cell HCC, H-E, x200; **c.** Macrotrabecular massive HCC, H-E x200; **d.** Scirrhous HCC, H-E x100.


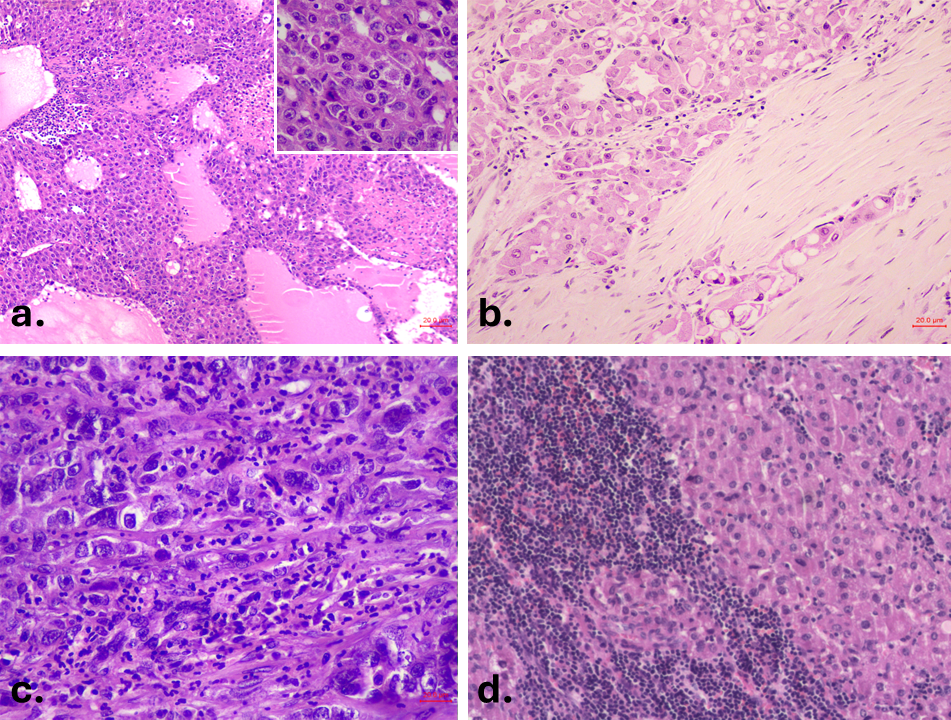
**Supplementary Figure S2**. **a.** Chromophobe HCC, haematoxylin-eosin (H-E) x50. Inset shows abrupt anaplasia, H-E x400; **b.** Fibrolamellar HCC, H-E, x200; **c.** Neutrophil-rich HCC, H-E x400; **d.** Lymphocyte-rich HCC, H-E x200
